# Cancer- and Non-cancer Related Chronic Pain: From the Physiopathological Basics to Management

**DOI:** 10.1515/med-2019-0088

**Published:** 2019-10-17

**Authors:** Antonello Sica, Beniamino Casale, Maria Teresa Di Dato, Armando Calogero, Alessandro Spada, Caterina Sagnelli, Mario Santagata, Pietro Buonavolontà, Alfonso Fiorelli, Anna Salzano, Concetta Anna Dodaro, Erika Martinelli, Elisabetta Saracco, Teresa Troiani, Dario Tammaro, Fortunato Ciardiello, Alfonso Papa

**Affiliations:** 1 Department of Precision Medicine, University of Campania Luigi Vanvitelli, Naples, Italy; 2 Department of Pneumology and Tisiology, AO Dei Colli - V. Monaldi, Naples, Italy; 3 Pain Department, AO Dei Colli - V. Monaldi, Naples, Italy; 4 Department of Advanced Biomedical Sciences, University of Naples Federico II, Naples, Italy; 5 Department of Mental Health and Public Medicine, University of Campania Luigi Vanvitelli, Naples, Italy; 6 Multidisciplinary Department of Medical Surgery and Dental Specialties, University of Campania Luigi Vanvitelli, Naples, Italy; 7 Thoracic Surgery Unit, University of Campania Luigi Vanvitelli, Naples, Italy

**Keywords:** Pain, Chronic pain, Non-cancer pain, Cancer pain, Intrathecal therapy, chronic pain therapy

## Abstract

The prevalence of chronic pain is between 33% to 64% and is due to cancer pain, but it has also been observed in non-cancer patients. Chronic pain is associated with lower quality of life and higher psychological distress and depressive/anxiety disorders in patients without a history of disorder.

In this study we evaluated in clinical practice the effectiveness of the intrathecal pump in 140 patients who underwent pain therapy at our Center. These patients were consecutively enrolled from January 2010 to July 2018.

Follow-up was carried out over these eight years regarding the infusion modalities. Pain relief was obtained in 71 (50,7%) patients out of the 140 that experienced satisfactory pain control globally.

Intrathecal therapy is one of the best options for chronic severe refractory pain. The greatest advantage of this therapy is due to the possibility of treating the pain with minimal dosages of the drug, avoiding the appearance of troublesome side effects.

## 1 Introduction

Pain is a very complex physiopathological entity. It can take on different aspects both from a clinical and biological/ physiological point of view, and present itself as an epiphenomenon of a pathophysiological process, until it becomes a real pathological entity in its own right. Pain is defined as chronic when it is continuous for more than three tosix months [1]. It is a condition reported in 20% of people worldwide, in 15-20% of all physician examinations [2], and should receive more attention because a proper pain therapy is a human right [2,3]. The trigger for the development of chronic pain may be different in different situations. Inflammation causes inflammatory pain, while nerve injuries as the result of mechanical trauma (iatrogenic or not), metabolic or autoimmune disorders, and cancer and chemotherapy may give neuropathic pain [4, 5, 6]. The excitation of the primary neurons due to prolonged inflammation induce a pathological response that persist beyond the period of recovery of the tissue, constantly stimulating the nociceptive pathways and thus generating chronic pain with changes in ion channels, receptors and nerve synapses. The distribution of neurotransmitters and neuromediators allows peripheral and central neurons to reach the depolarization threshold early to cause ectopic discharges to amplify and activate nearby cells, with chronic pain [7]. Neuronal pathophysiological mechanisms are integrated with immunological response, and neuropathic pain is considered a neuro-immune disorder [8, 9, 10, 11, 12, 13]. In fact, patients with complex regional pain, peripheral neuropathy and neuropathic pain associated with spinal cord injury syndrome have increased serum IL-4, IL-6 and TNF-α, as well as reduced serum IL-10 levels [14, 15]. The serum increases of IL-1β, IL-6, IL-2, TNF-α, and IFN-γ increase the intensity of chronic pain [16, 17, 18, 19]. Therefore, inflammatory and allogenic processes are supported by a complex balance between cells and cytokines (due to both pro and anti-inflammatory molecules) and the nervous system.

IL-6, TNF-α and IL1-β, produced by macrophages, are cytokines that amplify chronic pain. According to some studies, the administration of TNF-α is associated with thermal hyperalgesia, mechanical allodynia and hypersensitivity associated with pain. In the electrophysiological field, TNF-α can also increase the conductivity of K+ ions of the membrane in a non-voltage dependent manner, resulting in overall neuronal hyperexcitability and therefore neuropathic pain. TNF-α induce the release of glial mediators TNF-α-induced glial mediators cause endocytosis of GABA receptors with consequent reduction of inhibitory modulation of the GABAergic system. Longterm potentiation (LTP) is a physiological mechanism for strengthening a neuronal circuit and is involved in numerous nerve functions. Intrathecal therapy (IT) is a good choice to improve therapeutic results in chronic pain.

The aim of this study was to evaluate in clinical practice the effectiveness of the intrathecal pump in 140 patients consecutively enrolled from January 2010 to July 2018 who underwent pain therapy at our Center.

## 2 Materials and methods

One hundred and forty patients who underwent pain therapy at our Center were consecutively enrolled from January 2010 to July 2018. All procedures conducted in the study were in accordance with international guidelines, with the standards of human experimentation of the local Ethics Committees and with the Helsinki Declaration of 1975, revised in 1983.

At the baseline visit, each patient signed their informed consent for the use of their data in clinical research.

All 140 patients underwent complete physical examination. Their pain was evaluated with the McGill Pain Questionnaire (MPQ), recording the Numerical Rating Scale (NRS) of average pain, minimum pain, maximum pain and pain during exertion (scale 0–10: 0 = no pain, 10 = worst pain ever). All patients were evaluated for HBV, and HCV serum markers. Serum samples were tested for HBsAg, anti-HCV, total anti-HBc, and anti-hepatitis B surface antibody (HBs) using commercial immunoenzymatic assays (Abbott Laboratories, North Chicago, IL, USA: AxSYM® HBsAg (v2) M/S for HBsAg, AxSYM® HCV (v3) for anti-HCV, AxSYM® CORE™ (v2) for total anti-HBc, and AxSYM® AUSAB® for anti-HBs), as described in previous studies [20, 21, 22, 23, 24, 25, 26, 27, 28].

Epidemiological, clinical, and immunological data recorded at the baseline visit included patients’ age and sex and risk factors for the acquisition of pain as stated by the patients.

All cases were followed-up in these 8 years with complete physical examinations, laboratory tests were carried out and pain was re-evaluated.

## 3 Results

The demographic and clinical data obtained at enrolment are shown in Table 1.

**Table 1:** Demographic and clinical characteristics of the 140 enrolled patients

**N. of patients**	140
**Males, N. (%)**	98 (70.0%)
**Age, years (M + SD)**	51.2 ± 8.2
**Caucasian, N (%)**	140 (100%)
**Etiology of the pain, N(%): *chronic cancer pain***	99 (71.0%)
***chronic non-cancer pain***	41 (29.0%)
**Therapy, N (%)**:	
***Morphine in primary infusion***	120 (85.6%)
***Ziconotide in primary infusion***	3 (0.2%)
***Baclofen in primary infusion***	11 (0.8%)
***Ziconotide plus morphine***	3 (0.2%)
***Bupivacaine plus morphine***	3 (0.2%)

The 140 patients were predominantly male: 98 (70%), with a media age of 51.2 years old (range: 42-63). Of 140 patients, 99 cases had been subjected to a system for the treatment of chronic cancer pain; 41 patients were diagnosed with non-cancer pain. In non-cancer pain patients, the main diagnoses were neuropathic or mixed pain, chronic, not responsive to conventional drug therapy and / or burdened by the incidence of significantly elevated side effects with high doses. Some patients were not responders to spinal cord stimulation (Table 1).

Morphine was administered as the first intrathecal continuous infusion in 120 cases, in three patients ziconotide was used alone as the primary infusion, and 11 patients received primary Baclofen infusion alone. Ziconotide and Bupivacaine are analgesic adjuvants to morphine. In three patients, Ziconotide had been used in association with morphine as a secondary infusion, and three patients had a secondary infusion of Bupivacaine used in association with morphine. The patient was then given an intrathecal continuous infusion of one of the following: ziconotide 2.5 mL (2.5 μg at 1 μg per mL) daily, morphine 0.1-8 mg daily, baclofen 250-1000 μg daily or bupivacaine 3-8 mg daily.

From the follow-up carried out over these 8 years regarding the infusion modalities and the titration of the described drugs, the following data concerning pain relief was obtained: 71 (50,7%) patients out of 140 experienced satisfactory pain control globally, settling on a percentage of pain relief variable from 50 to 70%.

Only one patient, followed by oncologist, showed pain relief less than 30%.

In patients on follow-up with baclofen infusion for a diagnosis other than chronic pain, a variable reduction of the painful physiological symptomatology in spasticity was observed.

## 4 Discussion

Intrathecal therapy is a good choice to improve therapeutic results in patients with chronic pain. In fact, the morbidity and mortality of IT opioids are lower than those of systemic administration of opioids. A trial on 6398 patients in over a period of ten years [29], showed that there weren’t deaths correlated with the IT opioid infusion. In the US, death from systemically administered opioids are about 19,000 a year. Intrathecal drug delivery (IDD) is potentially a safer option for the patient [30].The Food and Drug Administration (FDA) has approved two intrathecal pain relief drugs: morphine free of preservatives and ziconotide peptide. Intrathecal opioid administration may cause side effects (vomiting, nausea, itching, constipation, urinary retention and neuroendocrine dysfunction) [31]. The most serious adverse reaction reported is respiratory depression [32], which occurs with the administration of opioid IT therapy or restarting of IT opioids after stopping [33]. The risk may be higher with hydrophilic opioids, such as morphine, and in cases where other depressants of the central nervous system (CNS), such as benzodiazepines, are administered [34]. The Polyanalgesic Consensus Conference (PACC) has recently recommended testing opioid therapy in an outpatient setting with a conservative dose, as a single dose of 0.15 mg morphine produces respiratory depression (defined as a PaCO2 above 40 or a lower respiratory rate at 10 breaths per minute) [35, 36, 37]. Ziconotide is a non-opioid drug approved for IT [38, 39, 40, 41], with adverse events of the CNS reported (nausea, nystagmus, vertigo, dysmetria, ataxia, agitation, hallucinations and coma) [42]. Neuropsychiatric adverse effects can occur after many months of asymptomatic infusion and are the main reason for its suspension [43]. Although an increase in serum creatinine kinase is present in 40% of patients, this significant increase typically occurs in the first two months after initiation of ziconotide therapy and only three cases of rhabdomyolysis have been described. There is no antidote for the overdose of ziconotide IT. The use of intrathecal bupivacaine (usually in combination with morphine) is off-label and it has occasionally been associated with numbness and rarely with weakness [44]. The PACC of 2016 presented evidence-based recommendations on patient survival, pathological process and use of IT drugs. Regarding cancer pain, IT is frequently thought to offer reliable, safe and effective treatment [45, 46, 47, 48, 49, 50].

Regarding the algorithm for IT management in patients with cancer pain, the prognosis, the probable disease progression in anatomical regions, features of tumors and findings of peri-procedural imaging are useful considerations for the selection of the device. Patients with cancer pain deserve special attention, in terms of implementation of IT and selection of drugs, together with the sustainability of a regime. Therefore, patients are stratified into 3 main categories by stage of disease and life expectancy. In the last PACC update, IT is used to treat related/unrelated cancer pain, and chronic-severe refractory pain [51]. The focus was on the patient’s age, although age contributions are reflected in the sustainability of the therapy, which is addressed elsewhere in the recommendations [52,53]. The costs of this therapy and its safety are better than the use of systemic opioids [54, 55].

The need to resort to the continuous infusion of subarachnoid drugs occurs when pharmacological, neurostimulation or surgical therapies have been unsuccessful. The main indications are: chronic non-cancer pain with a strong neuropathic component; chronic cancer pain with more than 6 months of life expectancy, and spasticity of degenerative and traumatic origin.

The procedure is completely reversible, the catheter can be removed if the expectations in terms of reduction / disappearance of the symptomatology are not met; the greatest advantage is the possibility of treating the symptoms (pain, spasticity) with minimal doses of effective drug, avoiding the appearance of troublesome side effects. Fixed-flow pumps have an indefinite duration, while programmable flow pumps are constrained to when the battery runs out, so they must be replaced before the battery expires.

The implant procedure involves positioning the catheter in the subarachnoid space, connecting it to the pump through a second catheter (extension) that is tunneled to the abdomen where the subcutaneous pocket containing the pump is packaged. Both the first and second phases of the operation are performed under local anesthesia, possibly paired with a mild analgosedation. In addition to the risks associated with the completion of a surgical procedure, the implants and / or the use of IT devices may also entail the following risks and complications: the displacement or disconnection of the catheter may occur; insertion of a catheter into the subarachnoid space may result in epidural bleeding, headache, hematoma, infection, spinal cord compression and/ or paresis; loss of cerebrospinal fluid (fluid that bathes nerve structures), with possible headache; persistent pain at the site of implantation of the catheter or pump; serum collection at the pump site just under the skin, which can be evacuated with a simple puncture; catheter migration, which can cause changes in the analgesic effect; allergic reaction or rejection of implanted materials;and pain localized at the implant site.

Our study demonstrates the effectiveness of intrathecal therapy in reducing severe chronic refractory pain compared to intravenous (i.v.) therapies. The importance of specialized staff dedicated to these procedures can drastically reduce adverse events and complications. It is a safe and manageable solution in pain management and can be considered for chronic refractory pain from all sources. Our data shows a longer follow up and case study than the others in the literature [56, 57, 58, 59, 60, 61, 62] and this reinforces our obtained results.

## 5 Conclusion

Chronic pain is correlated with lower quality of life and depressive/anxiety disorders (15.5%) in patients without a history of these disorders. Depression and anxiety are associated with more severe pain.

Intrathecal therapy is one of the best options in the treatment of chronic/severe refractory pain. The costs associated with therapy in terms of safety are significantly better than those of systemic opioids.

The greatest advantage of this therapy is due to the possibility of treating the pain with minimal dosages of the drug, avoiding the appearance of troublesome side effects. Non-responsive patients represent the ideal category to be addressed with “niche” techniques such as cordotomy.

Our results confirm that intrathecal therapy is one of the best choices in the management and treatment of severe chronic refractory pain. This therapy, overall, is safer than systemic opioids, which often need higher dosages to be effective. With systemic opioid therapy, the higher dosages and possible interferences with other drugs used in this type of complex patient allow an increase in the possibility of serious adverse events. Intrathecal therapy has demonstrated superiority in the management of dosages of these drugs which can be reduced to the minimum effective with this technique.

The use of minimum effective dosages of opioids in pain therapy allows a reduction in serious adverse events, and for this reason also a considerable reduction in the overall costs of treatment of chronic patients.

The need to resort to the continuous infusion of subarachnoid drugs occurs when pharmacological, neurostimulation or surgical therapies have been unsuccessful. The catheter can be removed calmly, if the expectations in terms of symptomatic reduction are not met.
